# The development of independent colleges and their separation from their parent public universities in China

**DOI:** 10.1057/s41599-022-01433-9

**Published:** 2022-12-03

**Authors:** Xu Liu, Yanli Zhang, Xiantong Zhao, Stephen Hunt, Wuyin Yan, Yitao Wang

**Affiliations:** 1 Centre for Higher Education Studies, Southern University of Science and Technology, Shenzhen, China; 2 School of Education, Shanghai Jiao Tong University, Shanghai, China; 3 Faculty of Education, Southwest University, Chongqing, China; 4 Department of Education, UCL Institute of Education, Practice and Society, London, England; 5 School of Education (Teachers College), Guangzhou University, Nanfang College Guanghzhou, Guanghzhou, China; 6 Faculty of Education, Soochow University, Suzhou, Jiangsu China

**Keywords:** Education, Business and management

## Abstract

With the highest number of students in private higher education in the world, China is updating the governance system for this sector. This process involves independent colleges, which were founded by and remain associated with public universities, but which are privately funded. As one of the strategies to improve the development of independent colleges, in 2008 the Ministry of Education asked these colleges to separate from their parent public universities. However, by the end of 2020, over half of the 2008 colleges had still not separated but, over the next year, the outstanding figure suddenly dropped by one-third. This paper analyses the factors affecting the separation from the viewpoints of different stakeholders. Private funders have an interest in making use of the parent universities’ resources and controlling and gaining financial returns from the colleges; for the parent universities, the management fees paid by the colleges are an important priority, while, for local government, more and better higher education places with less public finance is a key goal. Currently, there are few studies in the English language literature on independent colleges. By disseminating experiences of higher education reform in China, our findings could have important implications for government policymakers and for senior and practicing managers in universities.

## Introduction

Private higher education has become increasingly prevalent worldwide, accounting for about one-third of all HE enrolments (Altbach, 1999, 2016; Levy, 2018). In Asia, the two highest concentrations are in Japan at 75% of its HE institutions (Obunsha Education Information Center, 2021) and South Korea at 86% (Statistics Korea, 2021). China, with its 7.08 million students pursuing full-time studies in 773 private universities and colleges (called universities in general in this article), accounts for 28.58% of the whole of its higher education (HE) institutions (Ministry of Education, 2022), and has the largest number of students in private HE institutions in the world.

Globally, the definition of ‘private’ differs by social and cultural context. However, the registered legal ownership status in general can work as a criterion because a university cannot legally register as public and private at the same time (Cao, 2008). In China, private HE institutions do not receive public funds but are empowered to award a diploma or bachelor’s degree after 3/4 years of full-time study, respectively (National People’s Congress, 2017). These include *minban* colleges, independent educational institutions that are not a subsidiary of, nor belong to a public university: as Li notes, “[t]he term privatisation is avoided because it is considered politically incorrect in Communist China” (2013, p. 45). Independent colleges, privately run establishments with independent registrations, however, are affiliated with a parent public university (Liu, 2018): they are a formal form of HE organisation in which public universities cooperate with private forces to use market mechanisms to operate colleges (Feng, 2006). However, despite their name, these are neither private in a legal sense nor actually independent, having instead a significant public–private mixed composition. Their initial operational resources in terms of teaching and management were supplied by their parent universities, but their funding comes mainly from tuition fees charged directly to students.

**Fig. 1:**
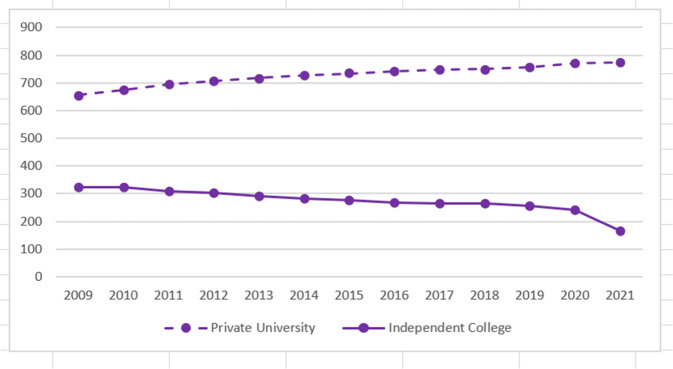
Private university and Independent College student numbers 2008–2021.

In 2008, the Ministry of Education (MoE) required independent colleges to separate from their parent public universities and transition into *minban* colleges: however, by the end of 2020, 240 (over 50% of the total number in 2008) had still not effected their separation. Figure 1 illustrates student numbers of private university and Independent College from 2008 to 2021. Why had many independent colleges not separated from their parent public university? But by the end of 2021, this figure had dropped dramatically to 160. What was the reason for this sudden change?

The severance of independent colleges’ relationships with their parent universities affects a number of stakeholders such as the government, parent universities and investors (Wang and Liu, 2021). Separation from the parent public university means that the cooperative relationship between private funders and the parent university is terminated. This may lead to conflicts of interest between the different stakeholders (such as what happens to the management fees paid by the independent college to its parent public university), even triggering legal disputes (Que and Zheng, 2021).

This study seeks to explore the factors affecting the separation of independent colleges from their parent public universities from the perspective of stakeholders. It first reviews the relevant literature and introduces the theoretical framework and the methodology, before going on to analyse government policy attitudes regarding independent colleges and how they facilitate the separation of independent colleges. It then discusses the interests of shareholders, the parent public university and the government, followed by the discussion and conclusion. There are only a few studies about independent colleges written in English. This article could have important implications for government policymakers, senior and practicing university managers, and academics by disseminating experiences of HE reform in China.

## Literature review

### The blurred division between private and public higher education

The public–private partnership (PPP) is an important concept in 21st-century discourses about governance. PPP is an arrangement whereby government states its need for capital-intensive, long-lived infrastructure and the desired facility is built using a complex combination of government, but mostly private, financing and then operated by a private entity under a long-term contract (Savas, 2000). The model of public universities working with private businesses is conducive to, for example, introducing private revenue streams (Savas, 2000), and many of the advantages and challenges of university–college partnerships are consistent with those in other sectors involved with PPP (Rosenau, 2000; World Bank, 2017).

Over time, the division between private and public HE has become increasingly blurred as HE has been progressively privatised by mounting private involvement in public universities (Fielden, 2010), the privatisation of services at public institutions, the corporatisation of universities, and publicly financed privatisation (Bjarnason et al., 2009), to the extent that public universities’ funds are deeply intertwined with those of the private sector (Ball, 2012). Universities often establish partnerships with private colleges that depend upon the public university’s supervision for a specified time or have to meet benchmarks before earning a separate institutional license (Levy, forthcoming). For example, in Ghana, new ‘university colleges’ have to be affiliated with a public university until they meet the criteria to become universities (Manuh et al., 2007). Such arrangements also have a long history in Latin America (Levy, 1986).

The state and public universities have been active in the creation of private HE institutions not only in China but also countries such as Russia (Suspitsin, 2007). Interestingly, this type of college is also common in India which has the highest number of higher education institutions (HEIs) in the world. In India, affiliated colleges usually focus on undergraduate education while universities usually offer post-graduate education (Masters and PhDs) and conduct research (Singh, 2003). In this way, the affiliation system allowed higher education to expand but, as the number of colleges and students grew, it also came under pressure in terms of maintaining quality and regulatory supervision (Sharma et al., 2013).

In China, HE PPPs began spontaneously before capturing the attention of and being endorsed by the government (Cai and Yan, 2009). The emergence of independent colleges was brought about by a number of pressures including the lack of public funding for public universities (Gu, 2006; Wen, 2011), the demand for HE exceeding the planned public supply (Ma, 2005), the investment interest of private enterprises, and families wanting their children to enter HE (Lai, 2004; Peng, 2013). The transformation of independent colleges’ status is realised not only in terms of a change in the external macroscopic institutional arrangement but also involves a series of internal and microscopic changes (Xia, 2021). Another key to a successful separation lies in whether the physical and structural conditions of the independent college, including campus size, meet the national standards attached to newly founded undergraduate universities (Yang et al., 2021; Zhong and Jing, 2021).

## The development of independent colleges

### Background

In 1999, the State Council initiated an HE enrolment expansion policy. However, the funds allocated for national education did not increase in proportion with the up-scaling of enrolment in HE. Minban colleges mostly only awarded 3-year diplomas, and thus did not appeal to those students who wanted to apply for 4-year bachelor’s degrees with their associated labour market advantage. Consequently, some public universities in more economically developed provinces channelled private funds to establish affiliated private colleges. Faced with this new mode of running colleges, the MoE decided to ‘wait and see’, which in government terms generally means tacit approval and encouragement, as this model appeared to be a viable means of dealing with the increasing demand for four-year HE bachelor’s degrees. The MoE’s attitude led to the rapid spread of this type of college from developed areas to most provinces in the country over the following few years.

By 2003, when the MoE issued its first landmark policy document, *Opinions on the Regulation and Strengthening the Management of Independent Colleges Sponsored Through New Mechanisms and Models by State Universities*, relating to this new type of college, there were already 360 in existence, all associated with public universities. As Levy (2006) points out, the delayed national regulations emerged in part to respond to this unanticipated growth in private HE. The *Opinions* termed these new institutions ‘independent colleges’, the first time that they had been called this in an official national document. The *Opinions* noted that some universities had made bold advances in the mechanisms of running HE, effectively operating relatively independent colleges. ‘Independence’ was cited in the document as a requirement for this type of college, in terms of campus, teaching facilities, teaching organisation and management, student enrolment and financial accounting. The aim of these regulations was to weaken the dependent relationship between the colleges and their parent universities.

While *minban* colleges educate students to diploma level, independent colleges dominate the undergraduate market, accounting for over 85% of all private college bachelor’s degree registrations. Applications to establish a bachelor’s degree programme and its associated student recruitment numbers have to be examined and approved by the MoE. As bachelor’s degrees are more advantageous in the job market, independent colleges, with their degree-awarding powers, are more likely to attract students in greater numbers and consequently realise a greater financial return than *minban* colleges.

### Cooperation between public universities and private sponsors

For independent colleges, there are two main types of co-sponsors that cooperate with public universities. The first—and most significant—are private enterprises or individuals: when public universities face a shortage of funds for expansion, many private firms take this opportunity to invest in HE and become involved with public universities in a cooperative running of the colleges (Liu, 2018). These can be divided into three categories. The first is retired government officials, university leaders and professors who have limited monetary capital but more social capital (Pan et al., 2013). They devote their personal energy and resources to running HEIs after retirement and their main goal is to promote the reform of the HE system by establishing private HEIs. The second category is made up of those whose goal is to obtain economic benefits from running HEIs (Wu, 2007; Wen, 2011), and the third is the social entrepreneur who hopes to contribute to society through running HE institutions (Jin and Wang, 2016). In fact, all these sponsors have different capitals and resources including political, economic, and social relationships with leaders of public departments such as educational administrative departments and universities.

The second category consists of state-owned enterprises: sometimes, in order to increase local HE resources, a city’s local government attracts reputed universities to establish colleges by providing free or heavily subsidised land and buildings. However, the national regulations for independent colleges do not allow local governments to be involved directly as they are public departments and publicly funded. In order to get around this restriction, local governments establish companies solely owned by local state-owned assets administration commissions, which act as a sponsor. As there is no policy that regulates whether this approach is allowed or not, many independent colleges have employed it in practice.

The regulations first stated that applicants entitled to found independent colleges should be public HE institutions that had bachelor degree awarding powers (MoE, 2003) although this was later changed to doctoral level (MoE, 2008). In other words, the government raised the requirements—and only around 15% of universities in China can award doctoral degrees, thus severely limiting the number of potential applicants. Independent colleges effectively splice public universities’ resources and private capital, making use of the advantages offered by public universities such as their social reputation and brand, employing their managers and teachers part-time, and also the flexibility of private companies’ social funds and management mechanisms. Historically, the most significant parallel is with existing universities creating regional university campuses, utilising the long-dominant HE mode to expand but without the quality and power risks associated with unrestricted new institutional entry (Levy, forthcoming). Once established, there is a formal hierarchical relationship between the independent college and its parent university, and the college pays a certain proportion of its tuition fees to the parent university every year.

## Stakeholder theory

Stakeholder theory provides the framework for this study. The word ‘stakeholder’ first appeared in 1963 in an internal memorandum at the Stanford Research Institute. This document defined stakeholders as including governmental bodies, political groups, financiers and investors, communities, employees, customers, and even competitors whose status is derived from their capacity to affect the corporation (Stewart et al., 1963). Clarkson (1995, p. 106) argues that stakeholders, “have, or claim, ownership, rights, or interests in a corporation and its activities”. Turker (2009) and Rake and Grayson (2009) provide examples of a wide range of stakeholders, including employees, customers, competitors, the natural environment, future generations, and governments. Freeman (2010) observes that a stakeholder is any group or individual who can affect or is affected by the achievement of corporate objectives. This importance of stakeholders is recognised by many organisations, including the OECD which states that

The competitiveness and ultimate success of a corporation is the result of teamwork that embodies contributions from a range of different resource providers including investors, employees, customers and suppliers, and other stakeholders. (2016, p. 8)

Thus the key issue is that the running of an organisation can be interpreted as being an interaction between the interests of its various stakeholders, and so institutions need to identify their stakeholders’ interests and shape governance actions toward satisfying them.

Mitchell et al. (1997) suggest defining the importance of stakeholders in terms of their different capacities and positions: power, legitimacy and urgency. In their opinion, stakeholders could have one or more of the following: (a) the power to influence the corporation; (b) a legitimate relationship with the corporation; and/or (c) an urgent claim on the corporation. Jamali and Mirshak (2007) concur; they acknowledge that although theoretically, all stakeholders are equally important, they differ in terms of the power, legitimacy and urgency they bring to the organisation. These different perspectives help to illustrate the roles of stakeholders and their relationships with each other.

**Fig. 2:**
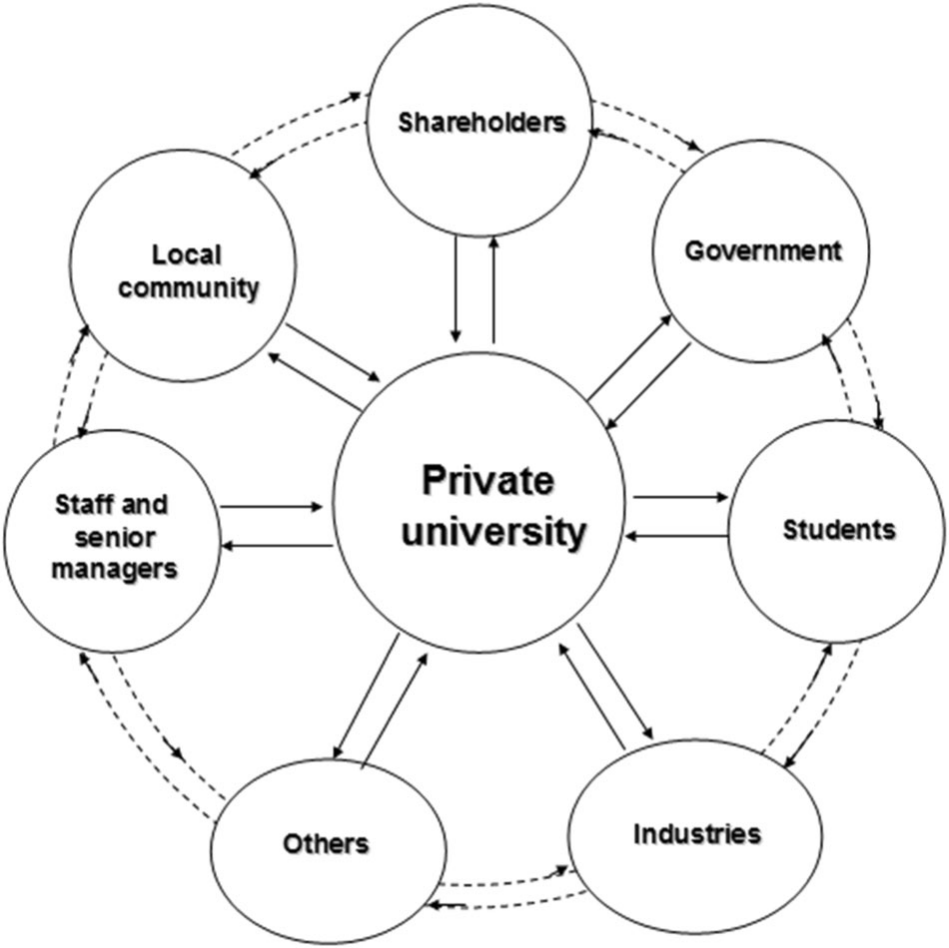
Stakeholder theory in relation to private universities.

With regard to universities, internal and external stakeholders can have different aims and values, each aiming to promote their own interests which may conflict with the interests of others (Ladd, 1975; Amaral and Magalhaes, 2002). Gillies (2011) expresses a similar opinion in that usually universities embody many competing interests, including students who want an education leading to a recognised qualification, staff members who want to progress in their career, the management team who seeks the best value for money, and governments which want to be re-elected. Of these, the students and the government are two key stakeholders (Cheng et al., 2021). Figure 2 lists stakeholders in private universities which can include students, employees, senior managers, shareholders, and policymakers (Liu et al., 2021).

Stakeholder theory, therefore, serves three purposes for this study. It first provides a perspective to recognise the different interests and actions of independent colleges’ stakeholders. Second, it highlights differences and possible conflicts arising from these differing interests for the college. Third, while the complexity of the environmental forces arises from the wider range of stakeholders and their impact on HE, the interests of its key stakeholders should be of prime importance in the governance of the university.

## Methodology

### Participants

The paper is part of a national research project on the governance of private universities in China. Interviewees came from three independent colleges located in two provinces. Two from Jiangsu Province, which has the most independent colleges of any province in China and is in the economically developed east, and one from Sichuan Province, which also has a large number of independent colleges, but is located in a relatively economically underdeveloped area in western China. Potential participants included stakeholders of the independent colleges including students, teaching and administrative staff, senior managers, shareholders, and policymakers (Liu et al., 2021). Before deciding who should be interviewed, we discussed this question with seven members of three independent colleges: three teaching staff, two administrative staff and two students. The teaching staff tended to be focused on work relevant to their subjects and students in their department and studies. Administrative staff had some understanding of the government policies but were not aware of the college leaders’ opinions of these policies.

As a result of these discussions, the final participants selected were independent college shareholders, senior managers and leaders of parent public universities; four officials from both the Sichuan and Jiangsu Provincial Departments of Education who understand the policies affecting independent colleges; three scholars researching private HE policy; and, two members of the Sichuan HE Provincial Review Committee.

All of them had held their current positions for at least three years and their average age was about 50; the oldest was 72 and the youngest 38. A snowball sampling technique was used in the recruitment of participants: the first three interviewees introduced more participants who introduced other interviewees. These participants all have rich experience in policy implementation and the issues affecting independent colleges.

### Procedure and materials

Fieldwork took place in 2020 but the COVID-19 epidemic severely compromised the original plan for face-to-face data collection, and the first round was finished online through WeChat. Interviewees were given an information sheet outlining the aims and process of the study before their interview and were informed that they could withdraw from the research at any time without having to give a reason. We visited the case study colleges in 2021 once the COVID-19 lockdown had been lifted and talked face-to-face with 28 interviewees, 16 of whom we had already talked to online in 2020 (see Table 1).

**Table 1:** Interviewee profiles and interview details.

	Position	Minutes	Location
*College A is located at Sichuan province*			
1.	President of College A’s parent university	40	Office
2.	President of College A	50	Online
		40	Office
3.	Shareholder A,	45	Online
		70	Office
4.	Shareholder B	70	Online
		40	Office
5.	Vice Secretary of the Communist Party Committee	70	Office
6.	Vice-President of Teaching	70	Office
7.	Vice-President of Finance	40	Online
		70	Office
8.	Vice President of Employment	40	Online
		60	Office
9.	Senior Advisor	50	Online
		60	Office
*Colleges B and C are located at Jiangsu province*			
10.	President of College B	90	Office
11.	Shareholder B, College B	40	Online
		40	Office
12.	Shareholder C, College B	60	Online
		60	Office
13.	Vice-President, Employment, College B	60	Online
		30	Office
14.	Head of the Student Development Department, College B	50	Online
		90	Office
15.	Head of the Development Planning Department, College B	50	Online
		90	Office
16.	President of College C	80	Online
		70	Office
17.	Shareholder A, College C	50	Office
18.	Shareholder B, College C	50	Online
		60	Office
19.	Vice-President, Teaching, College C	35	Online
		80	Office
20.	Senior Advisor, College C	90	Online
		90	Office
21.	Vice-President, Teaching, College C	90	Office
22.	Vice-Secretary of the UCPC, College C	70	Office
23.	Head of the Student Development Department, College C	60	Office
*Officials and researchers*			
24.	Official A, Jiangsu Department of Education	60	Home
25.	Official B, Jiangsu Department of Education	40	Online
		40	Office
26.	Official C, Sichuan Department of Education	40	Office
27.	Official D, Sichuan Department of Education	50	Office
28.	Member A of HE Provincial Review Committee in Sichuan	70	Office
29.	Member B of HE Provincial Review Committee in Sichuan	60	Office
30.	Private HE Researcher A	60	Cafe
31.	Private HE Researcher B	80	Office
32.	Private HE Researcher C	70	Online

Based on the trust and understanding developed during the first round, and subsequent face-to-face interaction, the informants provided useful information and insights on independent colleges. In August 2022, we added three more interviewees, one official from the provincial department of education and two scholars, to explore if there had been any significant changes in the research topic. Table 2 list quesions we used in the interview.

**Table 2:** Interview questions of the study.

1. How many years have you been working in the field of private higher education? What positions have you held?
2. Who do you think are the main stakeholders of an independent college?
3. Who has the most say in the separation of independent colleges from their parent public universities?
4. The Ministry of Education has been asking independent colleges to separate since 2008. After so many years, why have so many colleges still not separated?
5. What are the interests of private funders in independent colleges?
6. What are the interests of parent public universities in independent colleges?
7. What are the needs of the central and provincial governments? Are their interests the same?
8. Why did the number of separated colleges increase in 2021?
9. What do you think are the three biggest changes in your college since its separation from its parent university?

In order to protect their anonymity, names have been replaced with letters when referring to individuals and institutions. It is acknowledged that participants from three colleges in two provinces cannot represent all independent colleges in mainland China. However, the participants chosen have experienced the development of independent colleges, understand the regulations issued by the MoE and are knowledgeable about how independent colleges have developed in practice. This small but diverse body of participants allowed the study to concentrate on its particular objectives to illustrate the interests of different stakeholders. As such, the study provides an in-depth understanding of the factors affecting the separation of independent colleges from their parent public universities from the perspective of stakeholders.

This study also collected 31 documents, 17 at the regional level and 14 at the national level, from official sources for publications or websites which provide context for the development of independent colleges and identify policies issued by the government. For example, national-level documents include *Setting-Up and Management Method of Independent Colleges* (MoE, 2008) and *Implementation Plan for Accelerating the Transfer of Independent Colleges* (MoE, 2020), and at provincial level *Opinions of Energetically Carrying Out the Development of Private Higher Education* (Sichuan Province, 2009) and the *Regulations of Jiangsu on Promotion of Private Schooling* (Jiangsu Province, 2017).

### Process of analysis

The qualitative analysis software Nvivo was used for the thematic analysis of both documents and the interview data. Analysis of the extensive national documentation involving the governance and reform of independent colleges from the end of 1990 to the present provided the background to national and provincial policies. As a rich source of qualitative data, documents can provide insight into practices (Reischl and Plotz, 2020) and offer further information related to the contexts being explored (Schmoelz, 2020).

The study’s 44 interviews (31 participants, some of whom were interviewed twice) produced a total of 45 h of dialogue. Using Nvivo, the dataset produced a clear coding structure and a number of themes. The first step generated 58 codes which were combined to generate 20 themes, and then 11 categories. In the process of generating the categories, we re-familiarised ourselves with the transcripts and reflected on questions related to the research. For example, we asked, “What affects shareholder decisions?” “What are the interests of government and parent public universities” “How do these interests influence policy implementation?”, etc. These questions also guided us in coding the transcripts.

Table 3 shows the themes from the interview data. Eleven clearly distinguishable categories were identified and were then assigned to one of three analytical levels: (a) the development of independent colleges in China; (b) the challenges and policies facing independent colleges; and, (c) the interests of different stakeholders.

**Table 3:** Themes relating to data analysis.

Themes	Categories
Expansion of HE	The development of independent colleges
Lack of places in public universities	
Advantages offered by public universities	
Dependence on public universities	
Lack of independent resources for operation	
Purpose of the government	MoE required separation
Independence of independent colleges	
Challenges Policies	
Separations from public universities	
The interests of private funders	The interests of different stakeholders
The interests of parent universities	
The interests of the government	

## Different interests of key stakeholders

Stakeholders in independent colleges may have different interests and so be differently motivated about whether or not to re-register as an independent university quickly or prefer to delay the decision for as long as possible, maintaining the status quo. Among the most influential stakeholders are the provincial government, parent universities, and investment enterprises. Based on their different interests, these stakeholders compete over how the separation from the parent university, required by the central government, should be implemented. One example illustrates the interests of different stakeholders.

In the early days of X College, it was a professional training institution. The college was recognised by the market. However, because it did not have the qualifications to issue HE diplomas, it was difficult for it to expand or increase its tuition fees. In the end, we thought of a way to cooperate with a public university. At this time, the public university was concerned about raising funding for its own operation and development. So this professional training institution cooperated with the public university to establish X College of this public university. (Official B)

This interview shows the initial interests of the private institution and the public university. Another interviewee also talked about the process.

The public university is responsible for awarding HE diplomas and degrees to students and teaching the general education curriculum while the training institution is responsible for professional education, daily management and campus construction. The public university reported the cooperative agreement to the provincial department of education which neither agreed nor disagreed. The university guessed that meant that they approved the agreement. The school funding mainly comes from the tuition fees, of which 30% is handed over to the public university as management fees, and the remaining 70% is at the disposal of College C. (Shareholder A of College C).

The above statement illustrates the different interests of public universities and private institutions. The following explains the interests of the government, private funders, parent public universities in detail.

### MoE and incorporate the required separation

The most important characteristic of independent colleges is their dependence on their parent universities: some do not even have independent campuses, sharing all educational resources with their parent university, including libraries, laboratories and teaching facilities. These independent colleges usually have to transfer 15–40% of their tuition income to the parent university as the fee for their use of its reputation and educational resources. With limited educational resources of their own, many independent colleges often only offer the same subjects as their parent universities in order to get the maximum benefit from their situation.

These independent colleges offer popular majors such as art design, international economics and trade, business administration and accounting. (Vice-President of College C)

This leads to a lack of differentiation among independent colleges, making it difficult for them to form an independent character, which in turn affects their sustainable development. In order to address these challenges, the MoE (2006) asked independent colleges to gradually separate from their parent universities. This was followed, in 2008, by a number of requirements, including that they:

- be independent of the parent university campus, teaching management and recruitment of students;
- award an independent degree certificate and have independent financial accounting; and,
- have independent legal person qualification and civil liability.

Thus, after their early regulation-free development, independent colleges were gradually brought into regulated development channels established by government policy. In 2009, the MoE required them to formulate detailed plans for their separations from the parent public universities, and, in 2011, to put their plans into practice. This was followed in 2018, by a request to make the change as soon as possible.

Table 4 lists a number of documents regarding independent colleges issued by the MoE from 2006 to 2020. The most obvious demand expressed in these documents was for their separation from their parent universities.

**Table 4:** Documents issued by the MoE regarding independent colleges, 2008–2020.

Year	Document	Key points
2006	Establishment of Higher Education Institutions During the Eleventh Five-Year Plan	Independent colleges could gradually separate from parent universities
2008	Setting-Up and Management Method of Independent Colleges	Establishes the seven ‘independences’ of independent colleges required by the government
2009	Notice on the Compilation and Application of the Five-Year Transitional Plan for Provincial Independent Colleges	All independent colleges required to formulate detailed plans for their separation from parent universities
2011	Setting-Up of Ordinary Institutions of Higher Education During the Twelfth Five-Year Plan	To progress the separation process as planned in 2009 and accelerate the preparation required
2018	Notice on the Setting-Up of Universities	To ask independent colleges to complete the separation process as soon as possible
2020	Implementation Plan for Accelerating the Transfer of Independent Colleges	All independent colleges should formulate a separation work plan by the end of 2020 and complete the separation process as soon as possible

In 2020, the MoE stated that ‘the transfer of independent colleges should be the main channel for establishment of [new] universities’ and that ‘all independent colleges should formulate a transfer work plan’ by the end of the year. The purpose of the document, although not explicitly stated as such, was to encourage all independent colleges to separate from their parent universities in a few years (Officials A and B, Jiangsu Department of Education).

The government wants the separation to take place soon because this way of running colleges would be a big challenge to the governance reform of private universities which aims to manage private universities through classification as either profit or non-profit making. (Officials B and C)

The updated 2020 policy offers three ways to proceed. The first, registering as either a for-profit or not-for-profit private independent university, is suitable for those independent colleges sponsored by private funds, which have clear ownership of college property rights. The second is to reregister as a public university. This is appropriate for independent colleges that are financially sponsored by foundations, enterprises or other subordinate institutions of their parent public university, or companies owned by the local government. If neither of these is feasible, then the third ‘option’ is closure.

### Private funders’ interests

Private funders’ interests, as shown in the interview data, differed depending on the independent colleges’ stage of development. In the first stage, the colleges were still small, and relied heavily on their parent universities for institutional and educational resources, while the parent universities invested their brand, teaching facilities and other resources to support the colleges’ development.

In this stage, the investors would not like the loss of resources from parent universities, including management and teaching, especially as it is much easier to recruit students by using the name of parent universities. (Shareholder A of College C)

Parent universities permitted to establish independent colleges usually have a relatively long history, good reputations, and are trusted by prospective students. The independent college would almost always pay its parent university a certain proportion of its tuition revenue as management fees as its student recruitment depends so heavily on its connection with the parent university. Any substantial loss of fee-paying students would probably deal a fatal blow to an independent college given their dependence on tuition fees for income.

The MoE regulations state that all investments in independent colleges shall be transferred from the investors into the name of the relevant independent college, thus giving it ownership of the original assets of the investing private enterprises. According to existing national regulations, independent colleges have the property rights of a legal person, and private enterprises have no right to use these assets. Thus, no assets of independent colleges can be used as security for a loan or lent to any other organisation. The inconsistency between the property rights of a legal person and the restrictions on what the private funders can use their assets for presents a dilemma for investors deciding whether to separate from parent universities.

In order to meet the requirement as an independent university, an independent college usually has to invest more funds to improve its running conditions, including teaching facilities and expanding the campus. But this additional investment will not necessarily bring new benefits to investors. (Shareholder B of College A)

These MoE policies deter private investment interest as they reduced the investors’ degree of control over assets while at the same time increasing their overall costs. In addition, the investors’ interests had no proper security, so their enthusiasm at this stage to re-register independent colleges as private independent universities were, to say the least, reduced.

In this first stage, due to their dependence on their parent universities, it is difficult for these independent colleges to meet the MoE conditions to re-register as a university (e.g. teaching facilities and infrastructure). Investors invest because they expect to see a financial return and the sponsors of independent colleges need to balance costs and financial return.

When to separate from the parent university is definitely up to the investors. After that, how to invest in and run the university, including the salary level of the staff, all these decisions will still be made by the investors. Certainly, the sponsors play a leading role in the running of the university. (Vice-President of College C)

As independent colleges developed over time, they established a market presence and their dependence on their parent universities gradually reduced. In some cases, even the value of the parent universities’ intangible assets dwindled over time. For these more mature colleges, who continued to pay parent universities part of their tuition revenue as management fees following their initial contracts, the appeal of separation was greater.

We separated from our parent university in 2016. It was a very difficult choice. After the separation from our parent university, the monies we used to pay to it were invested in campus construction and teaching facilities. (President of College A)

Parent universities are often unwilling to let independent colleges separate because they are important income sources. However, they are required to follow MoE requirements or take the chance of being publicly criticised.

The investors had planned to separate from the parent university since 2018 but the ‘breaking-up fee’ we have to pay to the parent university was too expensive. After the MoE document was delivered in May 2020, we immediately started the process and will formally separate from the parent university in December 2020. (President of College B)

Independent colleges’ continual dependence on parent universities could restrict their independence in the future, especially if the college had already built a reputation of its own and was recognised by parents and students. Also, separation from parent universities means the colleges no longer have to pay high percentage management fees to the public university and so have more operating funds which would further stimulate their vitality and improve their sustainable development.

### Parent universities’ interests

Public universities have invested many intangible assets in independent colleges, including their brands, teachers and management for which, as compensation, the colleges pay a certain (usually 15–40%) percentage of their tuition fees to the parent university. Considering the amount of income this represents, it is not surprising that parent universities are reluctant to agree to independent colleges separating.

Independent colleges have, on average, about 15,000 students. This means the income from a single independent college exceeds tens of millions RMB every year as the independent colleges always pay a certain percentage of the tuition fee [to their parent institution]. (Official B, Jiangsu Department of Education)

The management fees from an independent college are a great income for the parent university. No leaders of the parent university would like the separation to happen during their tenures as they might be criticised by the staff. (Shareholder B of College B)

In order to disincentivise independent colleges from separating, parent universities usually ask for a high break-up fee as compensation, and this often becomes an obstacle to the implementation of the separation policy.

The excessive break-up fee increases the pressure on the survival of independent colleges, and the quality of education and teaching will be affected by the limited funds, so the independent college would stop the application. (Shareholder C of College B)

The MoE (2020) document, however, is a powerful impetus as it states that parent universities must acquiesce to independent college applications to separate.

The requirements of the MoE this time are very clear that those who can be transferred must be transferred as soon as possible, and the documents have been issued to independent colleges and parent universities. (Private HE Researcher A).

The parent universities have no choice but to acquiesce as the MoE has made a direct request; non-compliance may lead to a variety of punishments, thus encouraging the parent universities and the independent college to reach an agreement about the break-up fees as soon as possible. The question then becomes how to negotiate the break-up fee.

The parent university wants to negotiate a good price based on previous contracts, and the independent college hopes to separate quickly. Now both sides are active, and each has its own needs. (Official A, Jiangsu Department of Education)

It should also be noted that, once an independent college has separated, it has the rights of an independent legal person and this change in status means that the parent universities lose their supervisory powers. Although the parent universities usually still receive a proportion of fees for three or four years during the transitional period—until all the students who had registered before the separation graduate—they no longer have any actual power over the investment in and running of the college. In addition, in law, the investors in independent colleges occupy an equal status to the parent universities. Once re-registered and separated, independent colleges would be unwilling to allow their ex-parent universities to ‘interfere’ in their running.

When separating the independent college from its parent university is in line with the goals and interests of key stakeholders, the process is significantly accelerated: the MoE (2022) reported that almost 70 independent colleges separated from their parent universities in 2021.

### Provincial government interests

In mainland China, provincial administrative regions come directly under the central government. While working within the broad framework required by the MoE, provincial education departments formulate regulations in light of local social and economic development needs (Liu, 2019). Thus, different local governments may have different attitudes to and formulate different policies regarding regulations affecting independent colleges.

Some provincial governments don’t want to facilitate the transferring of independent colleges because the provincial finances can’t afford it. (Vice-President of College A)

Under the current national policy, provincial public universities are funded by the local government. When there are educational financial shortages, some local governments consider independent colleges to be stable channels to raise funds through student tuition fees. Thus, in order to reduce the HE burdens on local public finances, they are reluctant to facilitate the transferring of independent colleges to a public college or university.

One of the main factors influencing the attitude of the government is if there is enough in the public budget for education. The local government has to carefully allocate its limited budget in different areas including infrastructure, transport, medical system and so on. (Official C, Sichuan Department of Education)

The local economic development level also plays an important part in how much provincial governments support the separation of independent colleges.

After the separation of independent colleges, some provincial governments allocated a special fund to the colleges to improve their facilities. Some provincial governments waived the independent colleges’ assets transfer fee which was often US$ several million. But our province could not afford either of these options as the local public finance could not support them. (Official D, Sichuan Department of Education)

Positive support from the local government arouses investor enthusiasm and plays a crucial role in promoting the separation of independent colleges. However, to have more and better HE places with less public finance is the interest of the provincial governments, particularly so for those in economically underdeveloped areas. Thus, the re-registration processes in each province can be greatly affected by local government policies and their available public funds for education which, in turn, are influenced by the province’s economic conditions.

The national policies were quite strict when they were made in the central government, but when these policies are implemented at the local level, they are just like ripples in the lake and they do not have much influence in the end. (President of College A’s parent university)

The national policy, when implemented in individual provinces, may not always perform as planned. Although the national documents regulate the approach, standard, and procedures of the transfer, its implementation involves land, planning, finance, taxation, personnel and other issues which involve multiple departments of local government—and the policies formulated and implemented by these different departments sometimes conflict with each other. Provincial governments’ attitudes, thus, have a strong influence on the re-registration process:

There is a lack of overall co-ordination between departments which seriously affects the separation process of independent colleges. (President of College C)

Thus, in addition to a lack of money, local governments may also lack feasible coordinated supporting policies and mechanisms. The vision and power of the leaders in charge of education can also affect policy implementation. If the province’s governor for education and head of the department of education have sufficient influence, the implementation of the policy goes more smoothly: for example, if the deputy governor is a member of the Standing Committee of the Communist Party in the province, then the implementation of education policy would attract more support and resources.

## Implications

This study contributes to HE governance and management by exploring the factors affecting the separation of independent colleges from their parent public universities from the perspective of various stakeholders in China. For private funders of independent colleges, the key interests are enjoying the resources of parent universities and controlling and gaining financial returns from the colleges. For the parent universities, the management fees paid by the colleges are their priority. For local government, more and better HE places with less public finance are the quest.

As public–private partnership ventures, independent colleges both provided income for public universities and a potential economic return for investors. They also satisfied the local government’s desire for more and better HE places with less public finance. In the process of implementing the policy separations, independent colleges often face tensions between their various stakeholders which negotiate with each other to maximise their own interests; some may even take actions that benefit their own interests while damaging those of others. The main factor that determines the implementation of the government’s policy on independent colleges is whether the interests of key stakeholders are satisfied. Without this, it becomes more difficult to realise the policy objectives.

The findings show that independent colleges first developed at the beginning of the 21st century and were only later subjected to direct government regulation. As we know, new institutions often lack social standing and can even be seen as contradictory to socially ingrained ones. As a new organisation, it is particularly important to have legal and social acceptability to have legitimacy in society. Without recognition of their right to exist, new institutions may falter due to a lack of acceptance (Giesecke, 2006). In the early stages of their establishment, independent colleges relied on their connection with their well-known parent public universities to gain social recognition and the acquiescence of the government. Those with authority formally or informally evaluate aspects of an organisation and exert varying degrees of influence on the overall levels of legitimacy (Slantcheva and Levy, 2007). For the independent colleges in this study, a series of documents later issued by the government on the one hand put forward regulations and requirements, yet on the other strengthened the legitimacy of independent colleges, leading to more social recognition that, as a valid type of private HE institution, independent colleges are essential components of HE in China.

In a world in which private forms are materialising in breathtakingly new and fast-growing ways, they often inhabit territories uncharted by state plans and steering, territories very much in the unproven market (Levy, forthcoming). The government eventually notes which types and roles thrive, and which perhaps should be curbed, and regulation follows; this speaks to a state role, but its delay probably speaks more to pluralism than to state planning (Levy, 2006). The imbalance between HE supply and demand, lack of public funds for education and continuous economic growth comprise the basic ecological environment from which independent colleges sprang. The implementation of the enrolment expansion policy and investment by private enterprises as well as the policies of the education administration further contributed to the development of independent colleges.

These colleges have evolved to match the changing social and economic contexts and the colleges different developmental phases. Their governance actions depend not only on the rights, roles and legitimacy of their stakeholders but also on the developmental needs of the colleges themselves. Managers should therefore take this into account when designing their governance actions in order to meet the interests of stakeholders. The research findings from the various stakeholders indicate that, given the network of competing interests, prior to 2020 many independent colleges were unable to implement the transformation policy. However, after the more stringent 2020 policy (MoE, 2020) was issued, a large number of colleges separated from their parent universities. Effective implementation of the policy requires a coordination of the interests of the relevant stakeholders: understanding their vested interests, strengthening the communication among them, and broadly considering their opinions to achieve a balance of interests, will allow an eventual outcome consistent with the prevailing policy requirements.

## Data Availability

The datasets generated during and/or analysed during the current study are available from the corresponding author upon reasonable request.
